# *Leishmania donovani* whole cell antigen delivered with adjuvants protects against visceral leishmaniasis in vervet monkeys (*Chlorocebus aethiops*)^☆^

**DOI:** 10.1016/S1674-8301(12)60002-5

**Published:** 2012-01

**Authors:** Joshua Muli Mutiso, John Chege Macharia, Evans Taracha, Michael Muita Gicheru

**Affiliations:** a Department of Tropical and Infectious Diseases, Institute of Primate Research, Karen, Nairobi 24481-00502, Kenya;; b Department of Zoological Sciences, Kenyatta University, Nairobi 43844-00100, Kenya.

**Keywords:** visceral leishmaniasis, *Leishmania donovani*, vervet monkey, sonicated antigen, adjuvants

## Abstract

In a previous immunogenicity and efficacy study in mice, montanide ISA 720 (MISA) was indicated to be a better adjuvant than bacillus calmette guerin vaccine (BCG) for a *Leishmania* vaccine. In the present study, we report the safety, immunogenicity and efficacy of *Leishmania donovani (L. donovani)* sonicated antigen delivered with alum-BCG (AlBCG), MISA or monophosphoryl lipid A (MPLA) in vervet monkeys following intradermal inoculums. Vaccinated and control animals were challenged with virulent *L. donovani* parasites and the parasitic burden was determined. Only animals vaccinated with alum-BCG adversely reacted to the inoculum by producing ulcerative erythematous skin indurations. Non-parametric ANOVA followed by a post test showed significantly higher IgG antibodies, and revealed the presence of lymphoproliferative and interferon gamma responses in both AlBCG+Ag and MISA+Ag as compared to the MPLA+Ag or other groups (*P* < 0.001). We conclude that *L. donovani* sonicated antigen containing MISA is safe and is associated with protective immune response against *Leishmania donovani* infection in the vervet monkey model.

## INTRODUCTION

Leishmaniasis, a clinically heterogeneous group of diseases, caused by infection with protozoa of the genus *Leishmania*, is one of the world's most important infectious diseases[1]. Visceral leishmaniasis or kalaazar is the most dreaded and devastating amongst the various forms of leishmaniasis[2]. The disease is fatal in almost all cases if left untreated[3],[4]. It may cause epidemic outbreaks with high mortality[5]. There is currently no vaccine against leishmaniasis in routine use anywhere in the world[6],[7]. A vaccine against different forms of leishmaniasis should be feasible considering the wealth of information on the genetics and biology of the parasite, as well as on the clinical and experimental immunology of leishmaniasis, and the availability of *Leishmania* vaccines that can protect experimental animals against challenge with different *Leishmania* species[8]. Clinical and experimental studies demonstrate that generation of an effective cellular immune response is required for protection against this disease[9]-[13].

The only successful intervention against leishmaniasis is inoculation using virulent parasites, a process known as leishmanization[14]. Leishmanization was traditionally practiced by directly transferring infectious material from cutaneous lesions to uninfected individuals. However, leishmanization was largely abandoned due to safety issues and immunosuppression, which resulted in reduced immune responses to childhood vaccines[15]. Currently, only one country, Uzbebekistan, employs the use of leishmaniazation, where a mixture of live and dead *Leishmania major* (*L. major*) as a vaccine is licensed for high-risk populations[16]. Leishmanization was replaced by first-generation vaccines which are comprised of killed parasites or live attenuated *Leishmania* parasites[17]. Live attenuated *Leishmania* vaccines have been used to differing degrees of efficacy. Studies have included use of irradiated parasites[18], parasites attenuated by use of temperature sensitivity[19], or chemical mutagenesis[20] among other physical methods of attenuation. Attenuation by parasite gene modifications has also been carried out and these parasites have been used in experimental vaccination studies mainly in the murine system. BALB/c mice immunized with *L. infantum* lacking one allele of the silent information regulatory 2 (SIR2) gene developed strong Th1 immune response and conferred high degree of protection against a virulent challenge[21]. Immunization of susceptible and resistant murine model with dihydrofolate reductase-thymidylate synthase (DHFR-TS) deficient *L. major* parasites induced substantial protection against a virulent *L. amazonensis*[22] and *L. major* infection[23]. Although the use of attenuated parasites is considered effective in inducing long-lasting subclinical infection important in creating immunity to virulent infection, major safety concerns such as reversion to virulence and contraindication of these vaccines in immunosppression and pregnancy states have limited their development[24]. Vaccine formulation with killed parasites is still the most attractive in terms of cost and safety[25]. However, such killed vaccines require formulation with appropriate adjuvant for induction of desired cellular immunity for effective control of leishmaniasis. Numerous preparations of killed *Leishmania* parasites have been tested, either alone or in combination with a variety of different adjuvants[26]. Killed parasite vaccines using an alum-precipitated autoclaved *L. major* given with bacillus calmette-guerin vaccine (BCG) adjuvant have shown promise as vaccines for visceral leishmaniasis and post kalaazar dermal leishmaniasis[27]. However, this vaccine needs to be given in combination with antimonial therapy for enhanced cure rates and reduced incidence of relapse[28]. The most recent clinical trials of first generation vaccines have demonstrated a good safety profile but have not conferred significant levels of protection for use as prophylactic vaccines. It has been indicated that an appropriate adjuvant is important for an effective vaccine against leishmaniasis[6]. The availability of hundreds of adjuvants has prompted a need for identifying rational standards for selection of adjuvant formulation based on safety and sound immunological principles for human vaccines. We previously indicated that montanide ISA 720 (MISA) was a more effective adjuvant than BCG for *Leishmania* killed vaccine in the murine system[29]. Other studies have indicated the successful use of alum plus BCG[30] and monophosphoryl lipid A (MPLA)[31] in the control of visceral leishmaniasis in the monkey and murine systems, respectively. The present report describes a study undertaken to evaluate the safety, immunogenicity and efficacy of *L. donovani* sonicated antigen delivered with MISA, alum-BCG or MPLA in the vervet monkey model of visceral leishmaniasis.

## MATERIALS AND METHODS

### *Leishmania* parasites

*Leishmania donovani (L. donovani)* strain NLB-065 was originated from the spleen of an infected patient in Baringo district of Kenya and was maintained by intracardiac hamster-to-hamster passage at the Institute of Primate Research, Nairobi, Kenya. A hamster splenic biopsy was cultured in Schneider's drosophila insect medium supplemented with 20% fetal bovine serum and 100 µg/mL of gentamicin at 25°C till stationary phase. Stationary phase promastigotes were harvested by centrifugation at 2,500 *g* (Servoll 6000D) for 15 min at 4°C. The pellet was washed three times in sterile phosphate buffered saline (PBS) by centrifugation. These parasites were used for antigen preparation and challenge.

### Preparation of soluble *Leishmania* antigen

*L. donovani* stationary phase promastigotes were harvested by centrifugation as described above. Harvested promastigotes were washed and sonicated at 18 kHz for five times at 45 sec each on ice, separated by intervals of 1 min. The sonicated material was snap frozen and thawed three times in liquid nitrogen for extraction of whole soluble protein. The parasite suspension was centrifuged at 10,000 *g* for 30 min at 4°C. Protein concentration of the supernatant was determined using Bio Rad protein assay kit (Bio Rad) and stored at -70°C until use. This antigen was used for coating ELISA plates for antibody assay.

### Preparation of formalin-fixed *Leishmania* antigens

For *in vitro* lymphocyte proliferation and cytokine secretion assays, *L. donovani* promastigotes were harvested at the stationary phase and washed three times in sterile PBS as described before. Parasites were fixed in 1% formal saline for 1 h and then washed three times in PBS as described above. Parasites were counted in haemocytometer counting chamber and resuspended in a concentration of 5×10^8^/mL in sterile PBS and stored at -70°C until required.

### Adjuvants and vaccine preparation

MPLA (InvivoGen, San Diego, CA, USA), MISA (Seppic, Paris, France), alum (Rehydragel HPA; Reheis, Berkeley Heights, NJ) and BCG (Serum Institute of India, Hadapsar, India) were used as adjuvants in this study. The vaccination antigen was prepared from *L. donovani* promastigotes. Stationary phase promastigotes were harvested as described before, counted and resuspended in 3 mL PBS at a concentration of 8×10^8^ promastigotes/mL. These promastigotes were freeze-thawed three times in liquid nitrogen and sonicated at 18 kHz for five periods of 45 sec each on ice, separated by intervals of 1 min. Each vaccine antigen (sonicated) dose was made from 1×10^7^ promastogotes. Vaccine dosages included 1 mg alum precipitated antigen plus BCG (50 µL) and sonicate mixed with 40 µL MPLA. MISA was used at an adjuvant: antigen ratio of 7:3 as per the manufacturer's instructions. All vaccines were reconstituted in sterile PBS.

### Vervet monkeys

Both young and adult vervet monkeys of both sexes were caught in the wild and quarantined for 120 d at the Institute of Primate Research, Karen, Nairobi, Kenya. During the quarantine period, the monkeys were monitored for *Mycobacterium tuberculosis*, gastrointestinal and parasitic infections. The animals were tested for antileishmanial antibodies against both *L. donovani* and *L. major* antigen by ELISA. The monkeys with negative antibody test result were selected for this study. These animals were housed separately in squeeze-back cages and maintained on commercial non-human primate meal, supplemented with weekly fruits and vegetables. Water was provided *ad libitum*. Institutional Animal Care and Use and Institutional Scientific resources and Evaluation Committee guidelines were strictly followed.

### Experimental protocol

*L. donovani* antibody – free adult vervet monkeys with a mean body weight of 3.4 kg were selected and divided into five groups of three monkeys each as follows: group 1, alum precipitated sonicate plus BCG (AlBCG+Ag); group 2, sonicate plus monophosphoryl lipid A (MPLA+Ag); group 3, sonicate plus montanide ISA 720 (MISA+Ag); group 4, sonicate (Ag) alone and group 5 non-vaccinated control (naive control). The experimental groups were vaccinated three times intradermally at d 0, 28, and 42. Between d 0 and 60 vaccinated animals were monitored for safety parameters including induration and erythema at the vaccination sites. On d 21 after the last vaccination, immune responses were determined and all animal groups were challenged with 2×10^6^ virulent *L. donovani* parasites intravenously through the femoral vein on d 63 following the initial vaccination. Parasite burden was assessed on d 103 post challenge in splenic impression smears by counting the number of infected macrophages per 1,000 cell nuclei using a microscope.

### Enzyme linked immunosorbent assay (ELISA)

Polystyrene micro-ELISA plates (Dynatech Laboratories, Sussex, UK) were coated overnight with 100 µL of soluble *L. donovani* antigen (10 µg/mL) diluted in bicarbonate buffer, pH 9.6. Excess coating buffer was flicked off and non-specific binding sites were blocked with 3% bovine serum albumin (BSA) in PBS for 1 h at 37°C. Unbound BSA was washed off six times with 0.05% Tween 20 in PBS. One hundred microlitres of diluted serum (1/125 in 1% BSA in PBSTween) samples were dispensed into the wells and incubated for 1 h at 37°C. Unbound serum was washed off six times as described above and 100 µL of 1/2,000 horse radish peroxidase conjugated goat anti-monkey IgG was added and followed by incubation for 1 h at 37°C. Unbound conjugate was washed off before adding 100 µL orthophenyldiamine substrate (OPD, Sigma, UK, final concentration 0.4 µg/mL) in the citrate buffer. The plates were incubated at 37°C in the dark for 30 min and optical density was read at 630 nm in a microplate reader (Dynatech Laboratories).

### Lymphocyte proliferation assays

Peripheral blood mononuclear cells (PBMCs) were prepared from venous whole blood by density centrifugation as described previously[32]. The cells were adjusted to 3×10^6^/mL in complete RPMI 1640 medium (GIBCO, Langley, OK, USA), which consisted of 10% fetal bovine serum (Flow Laboratories, Rockville, ML, USA), 2 mmol/L *L*-glutamine (Sigma Laboratories, Santa Fe, NM, USA), 100 µg/mL gentamicin (GIBCO, Langley, OK, USA), and 0.05 mmol/L 2-mercaptoethanol (Sigma Laboratories, Santa Fe, NM, USA). One hundred micolitres of cell suspension were distributed to each well of 96-well round bottomed microtitre plates (Nunc, Roskilde, Denmark). A 100 µL volume of either 5×10^6^/mL formalin-fixed *L. donovani* promastigotes or 10 µg/mL concanavalin A (Con A, Sigma Laboratories, Santa Fe, NM, USA) was added to the wells. Control wells received 100 µL of complete RPMI 1640 medium. Cultures were prepared in duplicate and incubated at 37°C in humidified atmosphere containing 5% CO_2_ for 5 d for *Leishmania* antigen cultures and 3 d for concanavalin A cultures. The cells were pulsed with 0.5 µCi of [methyl-^3^H] over the last 18 h and then harvested on fibre filter (Whatman International Ltd., Maidstone, UK). Incorporation of radionuclide into DNA was determined by liquid scintillation spectrometry. Proliferation was expressed as the stimulation index (SI). 
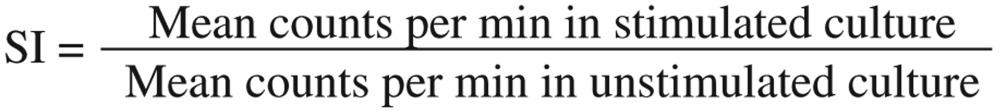


The SI values of the experimental groups were compared with the control monkeys. A SI value of > 2.5 was considered a positive response.

### Quantification of interferon-gamma (IFN-γ)

Purified PBMCs, were adjusted to 3×10^6^/mL in complete RPMI medium and stimulated with *L. donovani* promastigotes as described previously[33]. Culture supernatants were collected from triplicate wells after 72 h of stimulation, and the concentration of IFN-γ in the supernatant was determined by ELISA. Briefly, polysterene micro-plates (micro-ELISA, Dynatech Laboratories, Sussex, UK) were coated overnight with 50 µL of a 2 µg/mL concentration of capture monoclonal antibody to human IFN-γ (MabTech, Sweden) diluted in bicarbonate buffer (pH 9.6). Excess coating buffer was removed, and non-specific binding sites were blocked with 3% BSA (Sigma, Buchs SG, Switzerland) in PBS for 1 h at 37°C. The plates were washed four times with 0.05% Tween 20 in PBS, and 50 µL of culture supernatant was dispensed to appropriate wells. Human IFN-γ diluted (1 to 600 U/mL) in 1% BSA in PBS-Tween was used as a standard. The plate was incubated at 37°C for 1 h and then washed four times. Biotinylated secondary monoclonal antibody to human IFN-γ (50 µl of a 1/2,000 dilution) was added, followed by incubation at 37°C for 1 h. The plate was washed four times as before, 50 µL of 1/300-diluted alkaline phosphatase-conjugated streptavidin was added, and the mixtures were incubated for 1 h as described above. The plate was washed 10 times in PBS-Tween, and 50 µL of nitrophenyl phosphate substrate (1 mg/mL) in diethanolamine buffer was added. The plate was incubated at 37°C in the dark for 45 min, and absorbance was read at 405 nm. IFN-γ levels were assessed by comparison with the standard curve generated with human IFN-γ.

### Statistical analysis

Non-parametric one-way analysis of variance (ANOVA) was used to compare means of groups. Tukey-Kramer test was used for inter-group statistical analysis. Differences were considered significant if *P* < 0.05. Where applicable, Spearman rank correlation was used for correlation analysis.

## RESULTS

### Tolerability of the vaccine

All animals in the AlBCG+Ag group showed indurations at the sites of vaccination. These indurations, measured 7 d following immunizations, lasted between 34 and 41 d before complete resolution. These indurations were ulcerative and associated with erythema. Swollen lymph nodes were also observed in the AlBCG+Ag group and returned to normal size with the disappearance of indurations. Animals in the other groups did not show any lymphadenopathy or local skin reaction to the immunizations. There were no systemic side effects such as fever or weight loss in any of the animals groups following vaccinations.

### Anti-leishmanial antibodies

**Fig. 1:**
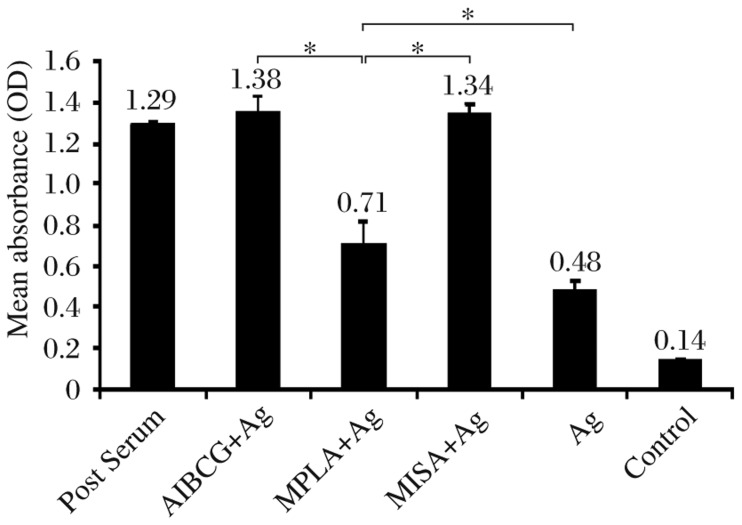
Anti-leishmanial antibody responses to *Leishmania donovani* in vaccinated animals. Animals were vaccinated at three different time points with sonicated antigen (Ag) alone or in conjunction with alum plus BCG (AlBCG+Ag), monophosphoryl lipid A (MPLA+Ag) or montanide ISA 720 (MISA+Ag) and anti-leishmanial antibody determined on d 21 after the third vaccination. Data shown indicate mean±SD in each vaccination group. Positive control serum from a previously infected vervet monkey with active *L. donovani* disease was included. *P < 0.001.

All vaccinated animals responded to *Leishmania donovani* antigen by producing IgG antibodies in levels ranging between (0.48±0.05) in the Ag group and (1.35±0.08) in the AlBCG+Ag group on d 21 following the second vaccine booster. Antibody responses in the AlBCG±Ag and MISA±Ag groups were comparable to the positive control serum and significantly higher than those in the MPLA+Ag group (*P* < 0.001). The MPLA±Ag produced significantly higher antibody responses than the Ag group (*P* < 0.001). There was no difference in antibody production in the control group as compared to the baseline values (***Fig. 1***).

### Lymphoproliferative responses to Con A and antigens

All experimental and control groups responded positively to Con A in the range between (237.54±23.76) and (205.00±74.55). The responses to Con A were comparable in all groups (*P* > 0.05). Lymphoproliferative response to the *Leishmania donovani* antigen was higher in the MISA+Ag group compared with the AlBCG+Ag group. However, there was no significant difference between these two groups (*P* > 0.05). Response to the antigen in the MISA+Ag was significantly higher than that in the MPLA+Ag vaccinated group (*P* < 0.001). The AlBCG+Ag vaccinated animals showed significantly higher response to the sonicated antigen than the MPLA+Ag vaccinated animals (*P* < 0.05). Although the Ag vaccinated group showed a slight positive response to the antigen, this response was not different from the control group (***Table 1***).

**Table 1:** Lymphoproliferative responses (stimulation indices)* in the vaccinated and control vervet monkey groups

Group	Stimulation index (SI)	
	Con A	Ag
AlBCG+Ag	218.29 ± 17.89	40.54 ± 3.2
MPLA+Ag	205.33 ± 74.55	19.41 ± 3.65
MISA+Ag	225.99 ± 43.71	52.40 ± 13.58
Ag	210.48 ± 7.77	2.62 ± 0.64
Control	237.54 ± 23.76	1.44 ± 0.06

^a^Responses were measured 21 d after the last vaccination.

* > 2.5 Stimulation index value to Con A/Ag was considered positive.

A1BCG: alum +BCG; MPLA: monophosphoryl lipid A; MISA: montanide ISA 720; Ag: antigen.

(mean±SD)

### IFN-γ response to Con A

**Fig. 2:**
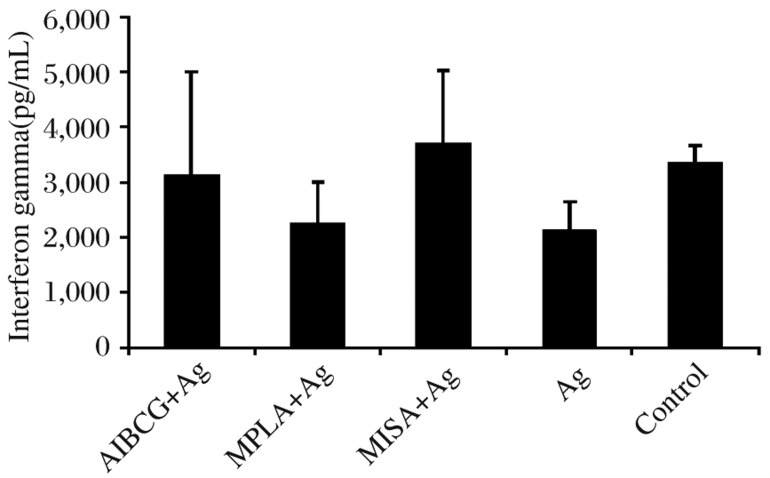
Level of IFN-γ in response to Con A. Animals were vaccinated at three time points with sonicated antigen (Ag) alone or in conjunction with alum plus BCG (AlBCG+Ag), monophosphoryl lipid A (MPLA+Ag) or Montanide ISA 720 V (MISA+Ag). Twenty one d after the third vaccination, PBMCs were stimulated *in vitro* with Con A before quantification of IFN-γ production 72 h later. Data shown indicate mean IFN-γ ±SD in response to Con A in each group.

Con A induced proliferation and production of IFN-γ in both the vaccinated and control groups as indicated in ***Fig. 2***. IFN-γ production in response to Con A showed wide variations within and between the groups but with no significant differences (*P* > 0.05).

### IFN-γ response to sonicated antigen

**Fig. 3:**
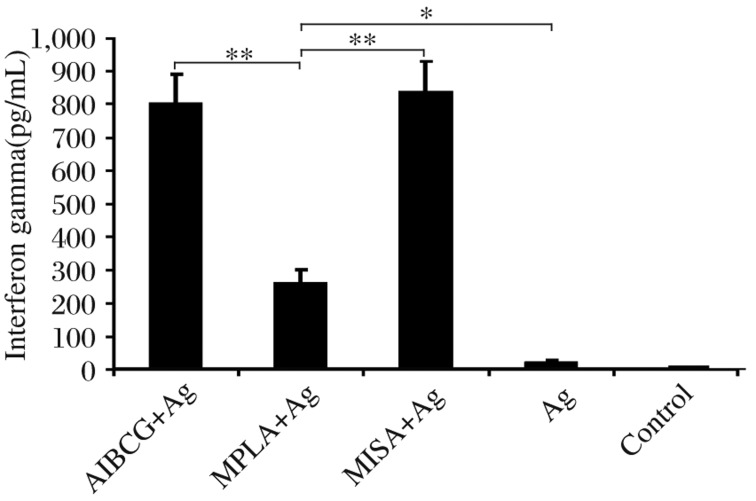
Level of IFN-γ in response to *Leishmania donovani* antigen. Animals were vaccinated at three time points with sonicated antigen (Ag) alone or in conjunction with alum plus BCG (AlBCG+Ag), monophosphoryl lipid A (MPLA+Ag) or montanide ISA 720 V (MISA+Ag). Twenty one d after the third vaccination, PBMCs were stimulated *in vitro* with *Leishmania donovani* antigen (Ag) and IFN-γ production was determined 72 h later. Data shown indicate mean±SD in response to Ag in each group. ^*^P < 0.01, ^**^P < 0.001.

When *L. donovani* antigen was used to induce secretion of IFN-γ, only the antigen plus adjuvant groups had detectable levels of the cytokine (***Fig. 3***). Animal groups vaccinated with either AlBCG+Ag or MISA+Ag induced the highest IFN-γ cytokine levels, which were comparable between the two groups (*P* > 0.05). These two groups induced significantly higher IFN-γ levels than the MPLA+Ag vaccinated group (*P* < 0.001). The MPLA+Ag group induced significantly higher IFN-γ levels when compared to the Ag or control group (*P* < 0.01).

### Assessment of parasite burden

**Fig. 4:**
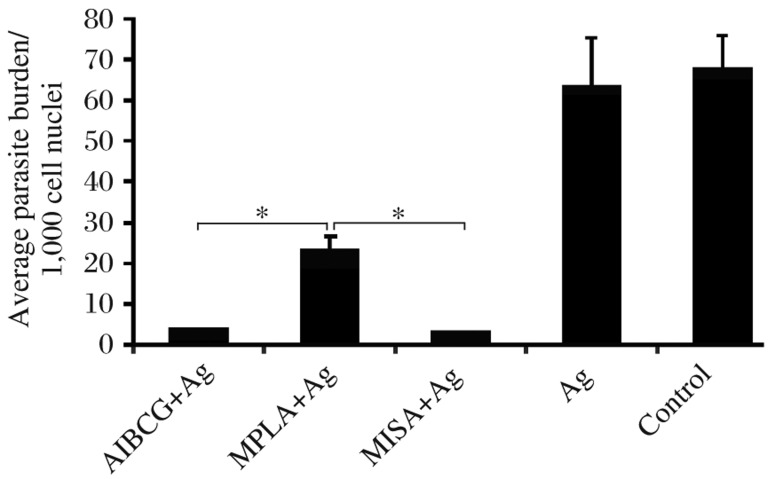
Parasitic burden in the experimental and control animals. Groups of vervet monkeys were vaccinated at three time points with sonicated antigen (Ag) alone or in conjunction with alum plus BCG (AlBCG+Ag), monophosphoryl lipid A (MPLA+Ag) or montanide ISA 720 V (MISA+Ag) and challenged with virulent *Leishmania donovani* promastigotes 21 d after the third vaccination. Parasite numbers were determined on Giemsa stained splenic impression smears on d 103 post challenge. Data shown indicate mean±SD per 1,000 cell nuclei in each group. ^*^P < 0.05.

A highly significant reduction in parasite burden was associated with the antigen-adjuvant vaccinated animal groups compared to the control group (*P* < 0.000,1). Comparably, highly significant reductions in parasite burden were associated with the AlBCG±Ag and MISA±Ag (*P* > 0.05). These two groups had significantly reduced parasite numbers as compared to the MPLA±Ag vaccinated group (*P* < 0.05). Parasite burden was comparable between the Ag and control groups (*P* > 0.05, ***Fig. 4***).

## DISCUSSION

In humans, recovery from *Leishmania* infection usually results in long-lasting immunity, indicating that vaccines against leishmaniasis are achievable. In this study, we used a nonhuman primate model to assess the safety, immunogenicity and protective capacity of a vaccine that combines *L. donovani* sonicated antigen delivered with either alum-BCG, MPLA or MISA as adjuvants. This kind of study was the first of its kind in this animal model. The results based on the safety parameters indicated that vaccination with MPLA+Ag and MISA+Ag was safe without any local or systemic side effects. The safety of MISA has been reported in earlier studies[34]-[36]. MPLA has also been proven safe in a vaccine study in healthy human toddlers[37]. Earlier studies had also indicated the safety and effectiveness of MPLA in *Leishmania* vaccine studies[38]. Additional safety evaluation of MPLA as an adjuvant for clinical trials have been done earlier in dogs, rabbits and rats[39]. However, as a matter of concern, alum-BCG vaccination was associated with erythematous ulcerative indurations and lymphadenopathy, which may preclude future use of BCG as adjuvant for *Leishmania* vaccines. Adverse effects of BCG have been reported before[29],[30]. Lack of systemic side effects, such as fever or weight loss in any of the vaccinated animals was encouraging considering the need for development of a safe vaccine for use in humans.

The association of AlBCG+Ag or MISA+Ag vaccination with high antibody levels was expected considering that these adjuvants are known to induce both cellular and humoral antibodies[34]-[36],[40]. We expected to get comparable antibody levels in the three adjuvant groups since MPLA is also an adjuvant associated with both cellular and high antibody responses[41],[42]. However, similarly to our results, a different previous study failed to get higher antibody levels in MPLA toddler vaccination compared to the control group[37]. Antibody levels were not predictive of disease outcome as antibody responses do not have any protective value in leishmaniasis. Furthermore, high antibody levels are associated with active visceral leishmaniasis infection[43]-[45].

Parasite-specific lymphocyte proliferation was demonstrated in all vaccinated animals with some variations in the magnitude of response. Marked Con A stimulation was demonstrated in all animals with no significant differences between groups. This was an indication of viability of the cells used in this assay. All vaccinated animals responded positively to *L. donovani* antigen, signifying the importance of this antigen in priming of lymphocytes in vaccinated animals, which may translate to strong T cell memory for long lasting immunity. The greater production of IFN-γ in AlBCG+Ag and MISA+Ag vaccinated groups than that in the MPLA+Ag group may indicate the superiority in potency of the former two adjuvants. High IFN-γ level has been considered one of the correlates of resistance[46],[47] and the cytokine is elevated in *L. major* self-cured animals[48]. The strong association with IFN-γ and resistance would be expected, since the parasites are killed when macrophages are activated by IFN-γ.

The ability to induce a protective immune response is the principal test of a new vaccine and adjuvant combination[35]. We have demonstrated that we were able to generate a cellular immune response that was sufficient to control parasite multiplication in the animal groups vaccinated with AlBCG+Ag or MISA+Ag. The high recall lymphoproliferative response and IFN-γ levels in these two experimental groups were predictive of disease outcome, and indeed, there was a highly significant reduction in parasite loads in the animals vaccinated with either AlBCG+Ag or MISA+Ag compared to other groups that had minimal interferon gamma levels. Failure of the MPLA+Ag used in this study to control disease to levels comparable to other antigen-adjuvant groups may be attributed to the formulation of this adjuvant. In a previous study using *Leishmania*-derived recombinant polyprotein Leish-111f antigen plus MPLA, protection against visceral leishmaniasis caused by *Leishmania infantum* was reported to be 99.6%[31]. However, the MPLA used in that study was formulated in stable oil emulsion while the MPLA used in our study was formulated in water. It appears that aqueous formulation of MPLA may be considered less effective than emulsion-based formulation[49]. However, in the study using toddlers, MPLA in aqueous formulation was associated with high cellular immune responses[37]. The difference in the toddler study with our results may be attributable to batch to batch disparities.

When considering the production of a *Leishmania* vaccine for clinical use, it would be desirable to produce a vaccine that is safe and able to control disease. Our present study shows that, of the two most immunogenic and protective antigen-adjuvant combinations (AlBCG+Ag and MISA+Ag), only MISA+Ag passed the criteria for a promising *Leishmania* vaccine in terms of safety, immunogenicity and efficacy. In a previous *Leishmania* vaccine study in the murine system, we observed that BCG vaccination produced unpleasant reaction that is undesirable for vaccination of humans[29]. The study recommended discontinuation of BCG as an adjuvant in *Leishmania* vaccines. Similar reports of the adverse effects of BCG in *Leishmania* vaccine studies have been mentioned in the India langur that used the intradermal route[30] for a vaccine against visceral *Leishmaniasis* in rhesus monkeys inoculated by the subcutaneous route[50] for a vaccine targeting cutaneous disease. Human *Leishmania* vaccine studies have also associated use of BCG adjuvant with ulcerative indurations[51]. Large ulcerating nodules as produced following vaccination with BCG may not be acceptable for field use of a vaccine.

Based on the present study, we report the suitability for use of MISA as a safe and effective adjuvant in the delivery of vaccines against visceral leishmaniasis in the vervet monkey model. The vervet monkey model has been well documented[35],[43],[46],[52] and these results may be evaluated in humans. In addition, MISA has been used in human vaccine trials[34],[36],[53],[54] and is strongly recommended by the manufacturer for clinical trials in humans[55]. We further recommend replacement of BCG with MISA as an adjuvant for Leishmania vaccines. However, considering that MPLA formulated in stable emulsion was shown to be highly effective in the control of L. infantum visceral leishmaniasis[31], we suggest a comparative study on the immunogenicity and efficacy between MPLA in stable emulsion and MISA in non-humam primate model of visceral leishmaniasis.
